# Bacterial inactivation by plasma treated water enhanced by reactive nitrogen species

**DOI:** 10.1038/s41598-018-29549-6

**Published:** 2018-07-26

**Authors:** Priyanka Shaw, Naresh Kumar, Hyong Sin Kwak, Ji Hoon Park, Han Sup Uhm, Annemie Bogaerts, Eun Ha Choi, Pankaj Attri

**Affiliations:** 1 Plasma Bioscience Research Center, Department of Electrical and Biological Physics, Kwangwoon University, 20 Kwangwon-Ro, Nowon-Gu, Seoul 139-701 Korea; 2 Research group PLASMANT, Department of Chemistry, University of Antwerp, BE-2610 Wilrijk-Antwerp, Belgium

## Abstract

There is a growing body of literature that recognizes the importance of plasma treated water (PTW) for inactivation of microorganism. However, very little attention has been paid to the role of reactive nitrogen species (RNS) in deactivation of bacteria. The aim of this study is to explore the role of RNS in bacterial killing, and to develop a plasma system with increased sterilization efficiency. To increase the concentration of reactive oxygen and nitrogen species (RONS) in solution, we have used vapor systems (DI water/HNO_3_ at different wt%) combined with plasma using N_2_ as working gas. The results show that the addition of the vapor system yields higher RONS contents. Furthermore, PTW produced by N_2_ + 0.5 wt% HNO_3_ vapor comprises a large amount of both RNS and ROS, while PTW created by N_2_ + H_2_O vapor consists of a large amount of ROS, but much less RNS. Interestingly, we observed more deactivation of *E. Coli* with PTW created by N_2_ + 0.5 wt% HNO_3_ vapor plasma as compared to PTW generated by the other plasma systems. This work provides new insight into the role of RNS along with ROS for deactivation of bacteria.

## Introduction

Over the last decade, several novel techniques have been developed for microbial decontamination^1,2^. Atmospheric pressure non-thermal plasma (NTP) has emerged as a potential tool for the effective control of photogenic microorganisms, and for water purification^3,4^. NTP generates ions, ozone (O_3_), UV photons, and various reactive oxygen and nitrogen species (RONS)^5,6^. It has shown promising effect in the sterilization of medical tools and packaging materials^7^. Recently, NTP has also shown its efficiency in wound healing^8–11^, tissue regeneration^12,13^ and cancer therapy^14–17^. Many types of plasma devices have been developed, such as dielectric barrier discharges (DBDs), plasma jets, floating electrode (FE)-DBD, and external additives have also been combined to the plasma treatment in order to increase the efficiency^14–16^. Previously, we have investigated the influence of H_2_O and D_2_O vapor to increase the efficiency of an atmospheric pressure plasma jet (APPJ) for the inactivation of cancer cells, i.e., breast cancer and melanocytes cancer cells^14,16^. In the last few years, plasma treated physiological fluids have also shown promising effect, similar to the direct treatment of plasma^17–19^. These plasma treated solutions contain reactive species with a longer lifetime, and provide a medium with a high and durable degree of reactivity^20^.

The chemical species generated in liquid treated by plasma mainly depend upon the type of discharge, feeding gas, humidity, etc. It was stated that plasma treated water (PTW) consists of various RONS, such as O (atomic oxygen), OH (hydroxyl radicals), O_3_ (ozone), H_2_O_2_ (hydrogen peroxide), NO (nitric oxide), NO_2_^−^ (nitrites), NO_3_^−^ (nitrates) and ONOO^−^ (peroxynitrites) that are responsible for microbial inhibition^21^. However, other authors conclude that short lived species are not stable in PTW, i.e., the life-time of excited atomic oxygen is ≈30 ns^22^, for OH it is ≈1 ns^23^, while O_3_ can live for 1000 s at room temperature^24^. It was also reported that reactive oxygen species (ROS), e.g. OH, O_2_^•−^, O_3_ and H_2_O_2_, play a significant role in bacterial inactivation^25,26^, whereas other research groups proposed that ONOO^−^ is the dominant species for bacterial inactivation^27,28^. Furthermore, it was reported in literature that the presence of NO_2_^−^/NO_3_^−^ at acidic pH in noticeable concentrations can cause the antimicrobial properties^29,30^. Traylor *et al*. discussed the complexity of reactions in PTW with biological systems, because different biological effects occur in differing time scales with various chemical components^25^. Finally, Ikawa *et al*. suggested that the main component for the antibacterial activity of PTW is not ONOOH (peroxynitrous acid), but O_2_NOOH (peroxynitric acid)^31^.

In general there is still uncertainty whether ROS or RNS are the main factor for the antibacterial effect in PTW. This paper aims to provide an answer to this research question about the importance of ROS and/or RNS in PTW for the antibacterial efficacy. Therefore, we developed an APPJ system with HNO_3_ vapor at different wt% and we checked the enhancement of NO along with OH radicals, both in the gas and liquid phase, using optical emission spectroscopy and chemical analysis, respectively. Besides NO and OH, the production of other reactive species, such as H_2_O_2_, NO_2_^−^ and NO_3_^−^, was also detected in solution after different types of plasma treatment (i.e., only N_2_ gas plasma, N_2_ + H_2_O vapor plasma, and N_2_ + 0.5 wt% HNO_3_ vapor plasma). Subsequently, we compared the antibacterial activity of PTW generated by the different plasma systems, by treating *E. coli*. Finally, we measured the oxidative stress related gene expression using quantitative real time polymerase chain reaction (qPCR) analysis, the genomic DNA degradation using gel electrophoresis, and changes in the surface morphology using scanning electron microscopy (SEM) of *E. coli* after treatment with PTW.

## Experimental Section

### Chemicals

Luria Bertani agar and broth were purchased from MB cells (Seoul, Korea). Strains of *Escherichia coli* (11775) were procured from the American Type Culture Collection, USA (ATCC). Penicillin–streptomycin was purchased from Gibco BRL (Carlsbad, CA, USA). Terephthalic acid and sodium hydroxide were purchased from Sigma Aldrich. Genomic DNA extraction and RNA extraction were done through a DNA extraction kit (GeneAll, Exgene Cell SV MAXI, Banseok Bld, Seoul, Korea) and a RNA extraction kit (RNeasy Mini Kit, Qiagen). cDNA synthesis was performed using the ReverTra Ace qPCR RT Master Mix with gDNA Remover kit (Toyobo, Japan), and quantitative PCR was performed using a Thunderbird Sybr® qPCR Mix kit (Toyobo, Japan).

### Plasma device and characterizations

**Figure 1:**
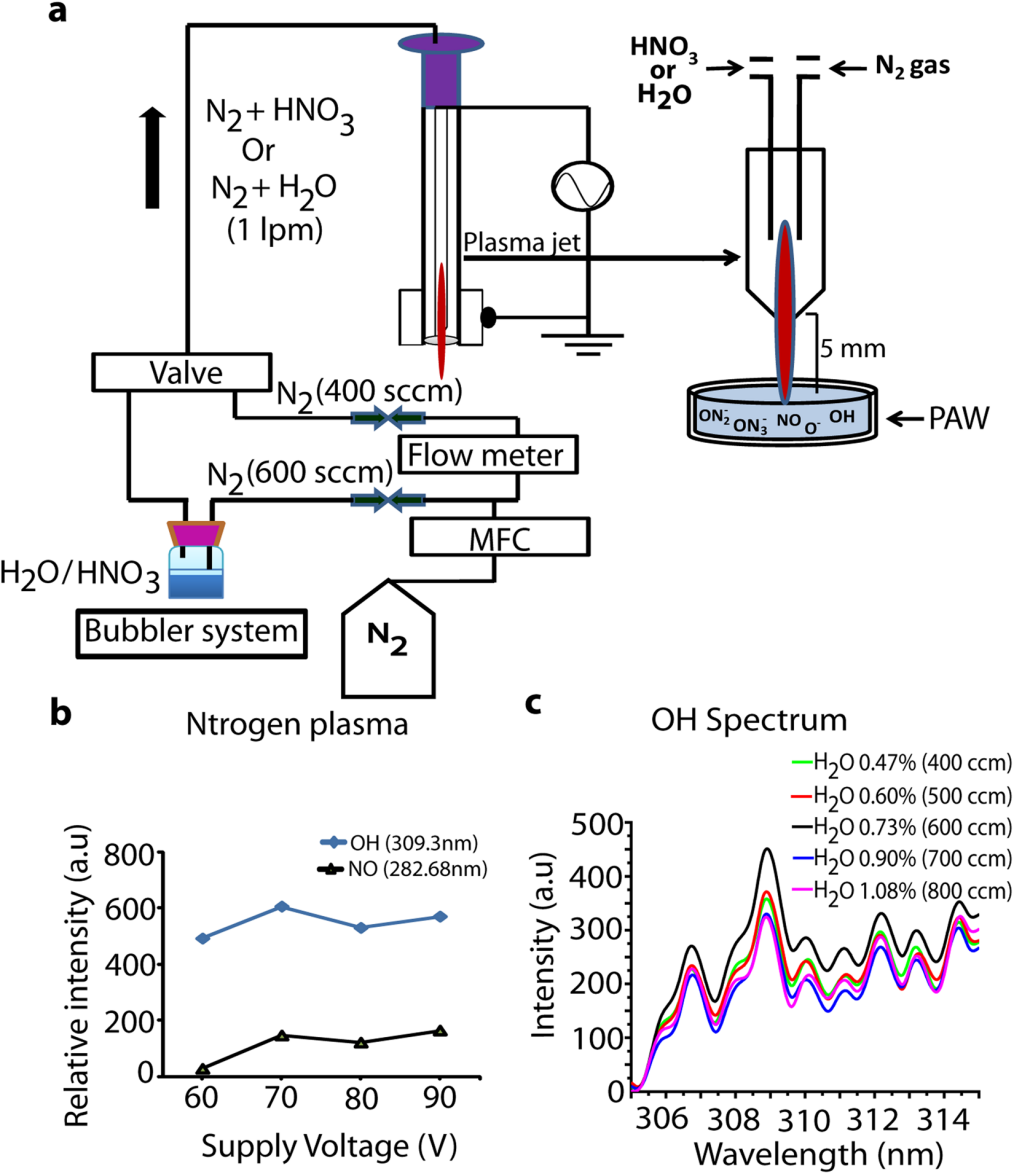
(**a**) Plasma setup; (**b**) Intensity of NO and OH spectral lines as a function of supply voltage in N_2_ plasma; (**c**) OH spectra for different gas flow rates.

The APPJ that was used consists of a needle-type powered (stainless steel) electrode that is enclosed by a quartz tube with inner diameter of 3 mm, outer diameter of 5 mm, and length of 9 mm. We used different flow rates of N_2_ gas in combination with different flow rates of H_2_O/HNO_3_ solution, so that the final gas flow rate remains 1000 cubic centimeters per minute (ccm). We applied an input voltage of 70 V, yielding an electrical power of about 7 W (2.2 kV, 11 mA with phase angle of 60° between current and voltage). The N_2_ gas flow rate directly introduced into the plasma was varied between 200 and 1000 ccm. Accordingly, the amount of N_2_ flowing through the solution of DI water with 0.5, 1 or 3 wt% HNO_3_, to generate H_2_O/HNO_3_ vapor that was fed into the plasma, also varied from 200 to 800 ccm. The combined gas (i.e., gas with vapor or without vapor) was injected into the needle of the plasma jet, and was flowing out through a 1 mm hole, as shown in Fig. 1a. Spectra of the APPJ emission were recorded by HR4000CG-UV-NIR (Ocean Optics, FL, USA) over a wide wavelength range (200–1100 nm), for a humidity of 40%. The electron temperature was measured from the emission spectrum using the SPEAC air program.

### Physical and chemical characterization of plasma treated water (PTW)

After exposing the water samples for 10 min to either N_2_, N_2_ + H_2_O vapor or N_2_ + HNO_3_ vapor plasma (at different wt% of HNO_3_, i.e., 0.5, 1 and 3 wt%), the pH and temperature of the sample were measured by a pH meter (Eutech Instruments, Singapore) and an Infrared (IR) camera (Fluke Ti100 Series Thermal Imaging Cameras, UK). Simultaneously, the RONS contents in the water samples were also analyzed. The amounts of OH and H_2_O_2_ were measured by previously described methods^32,33^. The concentration of NO_2_^−^ was measured using the Griess reagent kit (Molecular probe, USA) and the NO_3_^−^ concentration was measured with an Acorn ion Meter (Oakton WD-WD-35613-30 Ion 6 meter).

To prepare the PTW, 2 ml of DI water was treated with plasma for 10 min in a 12-well plate, keeping 5 mm distance between the nozzle of the plasma jet device and the liquid surface. To determine the colony forming unit, gene expression, cell morphology and DNA damage, we used 1 ml PTW generated by the different plasma conditions, i.e., N_2_, N_2_ + H_2_O vapor, and N_2_ + 0.5 wt% HNO_3_ vapor. The reason why we used the 0.5 wt% HNO_3_ vapor in our further experiments on the antibacterial efficacy (instead of 1 and 3 wt%) is explained below.

### Colony forming unit and disc diffusion assay

Strains of *Escherichia coli* (*E. coli*) were cultured in Luria broth (LB) media, until they reached the logarithmic growth phase of approximately 10^8^ colony forming unit (CFU)/ml. To determine the CFU, the culture was incubated for 12 h at 37 °C, and shaken at 200 rpm. Aliquots of the culture (100 µl) were pelleted by centrifugation at 5000 rpm for 5 min. The pellet was resuspended in 1 ml of PTW prepared by N_2_, N_2_ + H_2_O vapor, and N_2_ + 0.5 wt% HNO_3_ vapor plasma, to determine the antimicrobial effects. The suspension was incubated for 3 h at room temperature, and shaken at 200 rpm. We also performed direct plasma treatment, for which we took the same growth phase of *E. coli* in 1 ml of water, and directly exposed it for 10 min with either N_2_, N_2_ + H_2_O vapor, or N_2_ + 0.5 wt% HNO_3_ vapor plasma. Both the indirect and direct plasma treated samples were subjected to serial dilutions of 10^7^, 10^6^, 10^5^, 10^4^, and 10^3^ CFU/ml. The samples were thoroughly mixed, and a tenfold serial dilution of each sample was transferred and spread uniformly on LB agar culture medium in a standard Petri dish (90 mm). The samples were then sealed and incubated at 37 °C for about 12 h, to count the CFUs. We measured a relative reduction compared to the control sample (for which the CFU was defined as 1 to evaluate the inactivation efficacy.

In addition, we performed another experiment on Disc diffusion to assess the antibacterial activity of the PTW, for which the antimicrobial susceptibility testing was carried out according to the standard method by Bauer *et al*.^34^. The *E. coli* culture was evenly applied to LB agar culture plates using a sterile swab. The plates were dried for 15 min, and subsequently used for the sensitivity test. The discs that were impregnated with PTW produced by N_2_, N_2_ + H_2_O and N_2_ + 0.5 wt% HNO_3_ vapor were placed on the LB agar surface. One positive control, which was a disc, contained 30 µg of standard commercial antibiotic (Penicillin). The plate was then incubated at 37 °C for 18 to 24 hours. After incubation, the plates were examined for the inhibition zone.

### *E. coli* morphological analysis

Scanning electron microscopy (SEM) (JSM 7001 F, JEOL, Tokyo, Japan) was applied to examine the morphology of the *E. coli* cells. Briefly, the bacterial samples exposed to PTW generated by either N_2_, N_2_ + H_2_O vapor, or N_2_ + 0.5 wt% HNO_3_ vapor were fixed in 1 mL of Karnovsky’s fixative (2% paraformaldehyde and 2% glutaraldehyde) overnight, as described in previous work^5^. The SEM sample preparation involved dehydration of the material in hexamethyldisilazane (HMDS), followed by mounting and coating on glass with carbon tape, and examination via SEM.

### RNA extraction for quantitative real time PCR

To perform a quantitative evaluation of oxidative related gene expression, after 3 h exposure with PTW produced by N_2_, N_2_ + H_2_O and N_2_ + 0.5 wt% HNO_3_, the total RNA was extracted from treated and untreated samples of *E. coli* using an RNeasy Mini Kit, and it was converted to cDNA using reverse transcriptase and random primers (GoScriptTM Reverse Transcription System, Promega). The same amount of total RNA was used for the cDNA synthesis (Take3, Biotek). The resulting cDNA was used for the qPCR analysis (CFX96, Biorad) with primers (Macrogen) of 16 s rRNA (the RNA component of the small subunit used as house-keeping gene), OxyR, RpoE, GroES, and DnaK.

The primer sequences used for the oxidative related mRNA expression in E. coli were:

**Table:** 

Genes	Forward primers [5-3]	Reverse primers [5-3]
16S rRNA	AGAGCAAGCGGACCTCATAA	TTCATGGAGTCGAGTTGCAG
OxyR	GGGAAAACTGCTGATGCTG	CGCGGAAGTGTGTATCTTCA
RpoE	AGTCCCTCCCGGAAGATTTA	ACCTACCGGACAATCCATCCATGA
GroES	TGGCCGTATCCTTGAAAATG	CCGTAGCCATCGTTGAAAAT
DnaK	GAAGAAGCAGGCGACAAACT	TAGCGGCCTTTGTCTTCACCT

### DNA extraction for agarose gel electrophoresis

Genomic DNA was extracted after 3 h exposure with PTW produced by N2, N_2_ + H_2_O and N_2_ + 0.5 wt% HNO_3_ for 1% agarose analysis. The treated cells were subjected to a genomic DNA extraction kit. Genomic DNA was extracted following a standard molecular biology protocol, and re-suspended in 50 µl water. The same amount of genomic DNA (2 mg) extracted from *E. coli* was loaded on a 1% agarose gel, and run for 1 h. After staining with ethidium bromide, the DNA bands were photographed.

### Statistical analysis

All values are represented as the mean ± SD of the indicated number of replicates. Statistical analysis of the data was performed using the Student’s t-test to establish the significance between the data points, and significant differences are based on *P < 0.05 or **P < 0.01.

## Results

### Determination of NO and OH intensities at H_2_O vapor plasma condition

Figure 1(b) shows the NO and OH relative intensities during the plasma discharge, as a function of the applied voltage. Applying 70 V input voltage shows the high intensity of both NO and OH peaks measured through optical emission spectroscopy. Therefore, our further experiments were performed at an input voltage of 70 V. In order to evaluate the highest generation of OH radicals, we applied different flow rates of N_2_ gas in H_2_O solution, i.e., 400, 500, 600, 700, and 800 ccm, to generate the H_2_O vapor along with the N_2_ gas, and we examined the emission spectra of the plasma. Figure 1(c) shows that the OH emission peak was highest at 600 ccm of N_2_ gas flow.

### Determination of NO and OH intensities at HNO_3_ vapor plasma conditions

**Figure 2:**
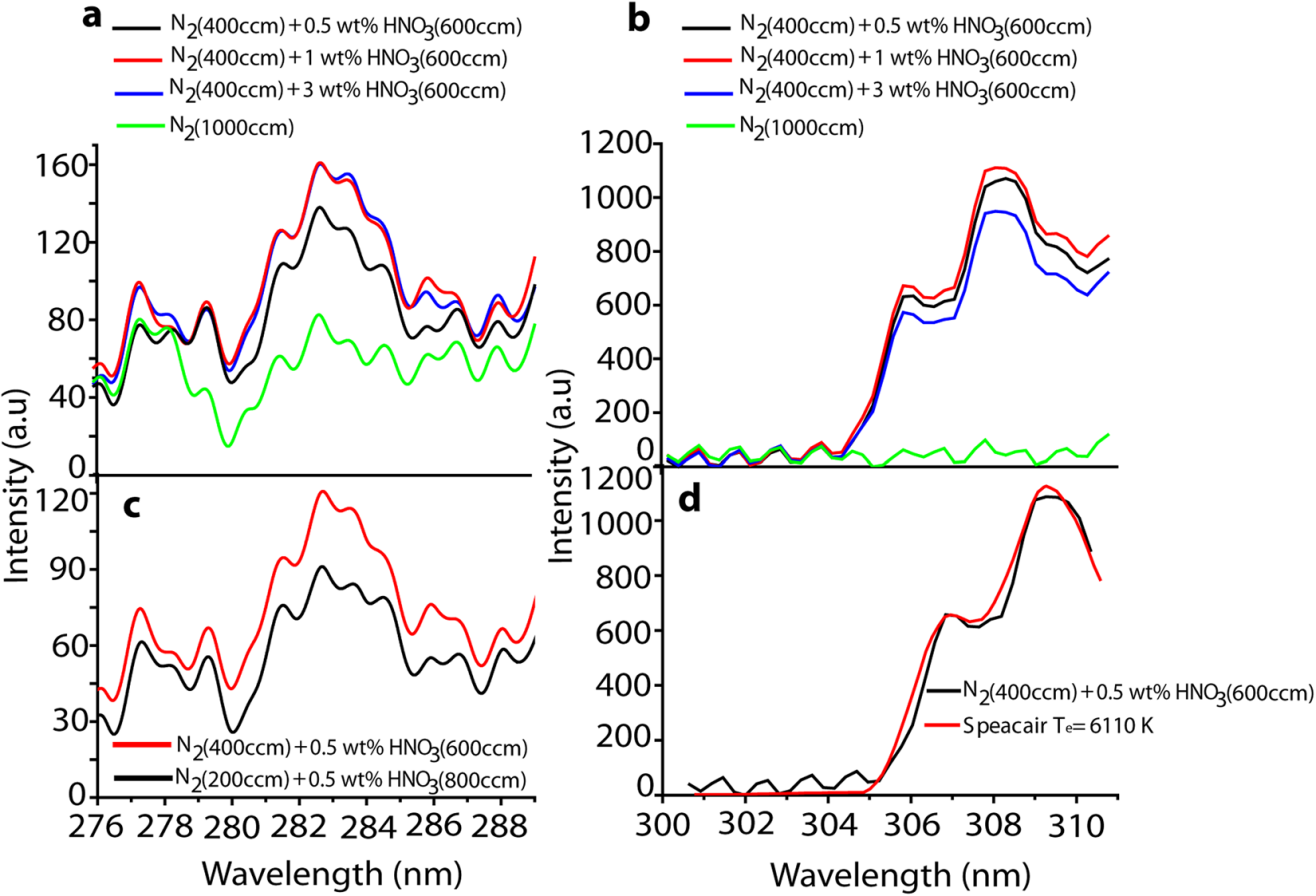
(**a**) NO spectra at different wt% of HNO_3_ vapor plasma, (**b**) OH spectra at different wt% of HNO_3_ vapor plasma, (**c**) NO spectra at different flow rates of N_2_ gas, (**d**) Determination of the electron temperature using speacair for the OH spectrum.

We applied different wt% HNO_3_ (0.5, 1 and 3 wt%) with a fixed N_2_ gas flow rate, and we also compared with no HNO_3_ vapor. Figure 2(a) shows that 1 and 3 wt% of HNO_3_ vapor yields a high spectral intensity for NO, whereas Fig. 2(b) shows that 0.5 wt% of HNO_3_ vapor yields a high OH intensity. The further experiments are performed using 0.5 wt% HNO_3_ vapor plasma, based on the change in pH of the solution after treatment (as described below).

We also analyzed the NO spectra for different flow rates of N_2_ + 0.5 wt% HNO_3_ vapor plasma. Figure 2(c) shows that N_2_ with 600 ccm added to 0.5 wt% HNO_3_ solution vapor, combined with 400 ccm of N_2_ gas, resulted in a higher NO intensity, in comparison with 800 ccm N_2_ added to 0.5 wt% HNO_3_ solution vapor, combined with 200 ccm of N_2_ gas. Finally, the electronic temperature in the plasma generated at N_2_ (400 ccm) +0.5 wt% HNO_3_ vapor (with 600 ccm N_2_ gas) was determined to be 6110 K using Specair, as shown in Fig. 2(d).

### Determination of pH and temperature after plasma exposure

**Figure 3:**
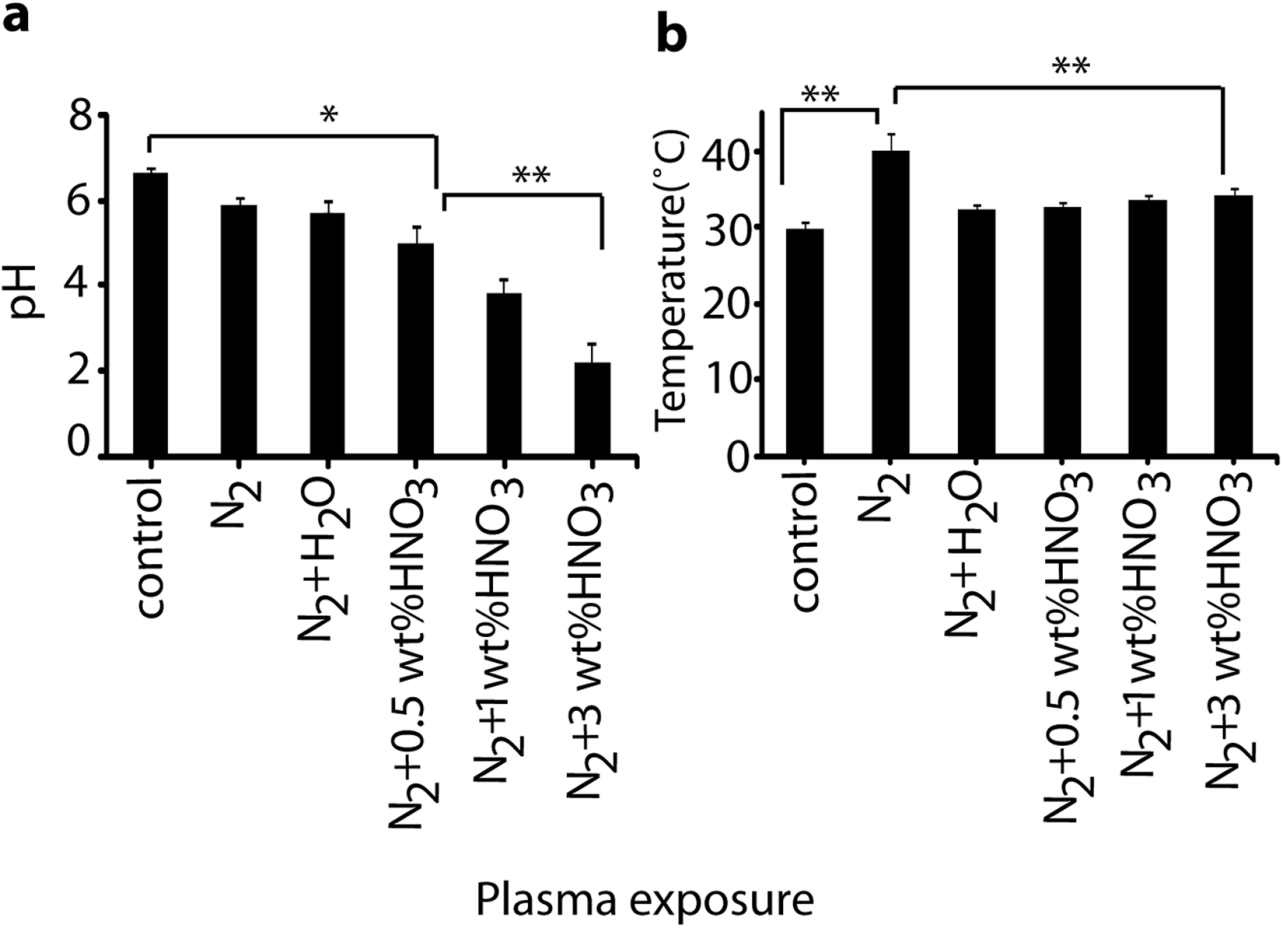
(**a**) pH and (**b**) temperature in DI water after exposure with plasma jet for 10 min, at different conditions: 1000 ccm N_2_ plasma, or 400 ccm N_2_ plasma with either H_2_O or HNO_3_ vapor at three different wt%, and comparison with untreated water sample (control).

To determine the change in properties of PTW after exposure with plasma, we checked the pH and temperature for different conditions, i.e., N_2_ (400 ccm) + (0.5, 1 or 3 wt%) HNO_3_ vapor (in 600 ccm N_2_ gas) plasma, N_2_ (400 ccm) + H_2_O vapor (in 600 ccm N_2_ gas) plasma, and N_2_ (1000 ccm) plasma without vapor. Figure 3(a) shows that 10 min treatment with plasma of N_2_ (400 ccm) + (0.5, 1 and 3 wt%) HNO_3_ vapor (in 600 ccm N_2_ gas) drastically decreases the pH, as compared with N_2_ + H_2_O vapor plasma and N_2_ plasma without vapor. On the other hand, there is only a slight rise in temperature (few °C) of PTW for N_2_ (400 ccm) + (0.5, 1 and 3 wt%) HNO_3_ or H_2_O vapor (in 600 ccm N_2_ gas) plasma, while there is a significant rise of about 10 °C for the N_2_ plasma without vapor, as shown in Fig. 3(b).

### Estimation of RONS contents in plasma treated water

**Figure 4:**
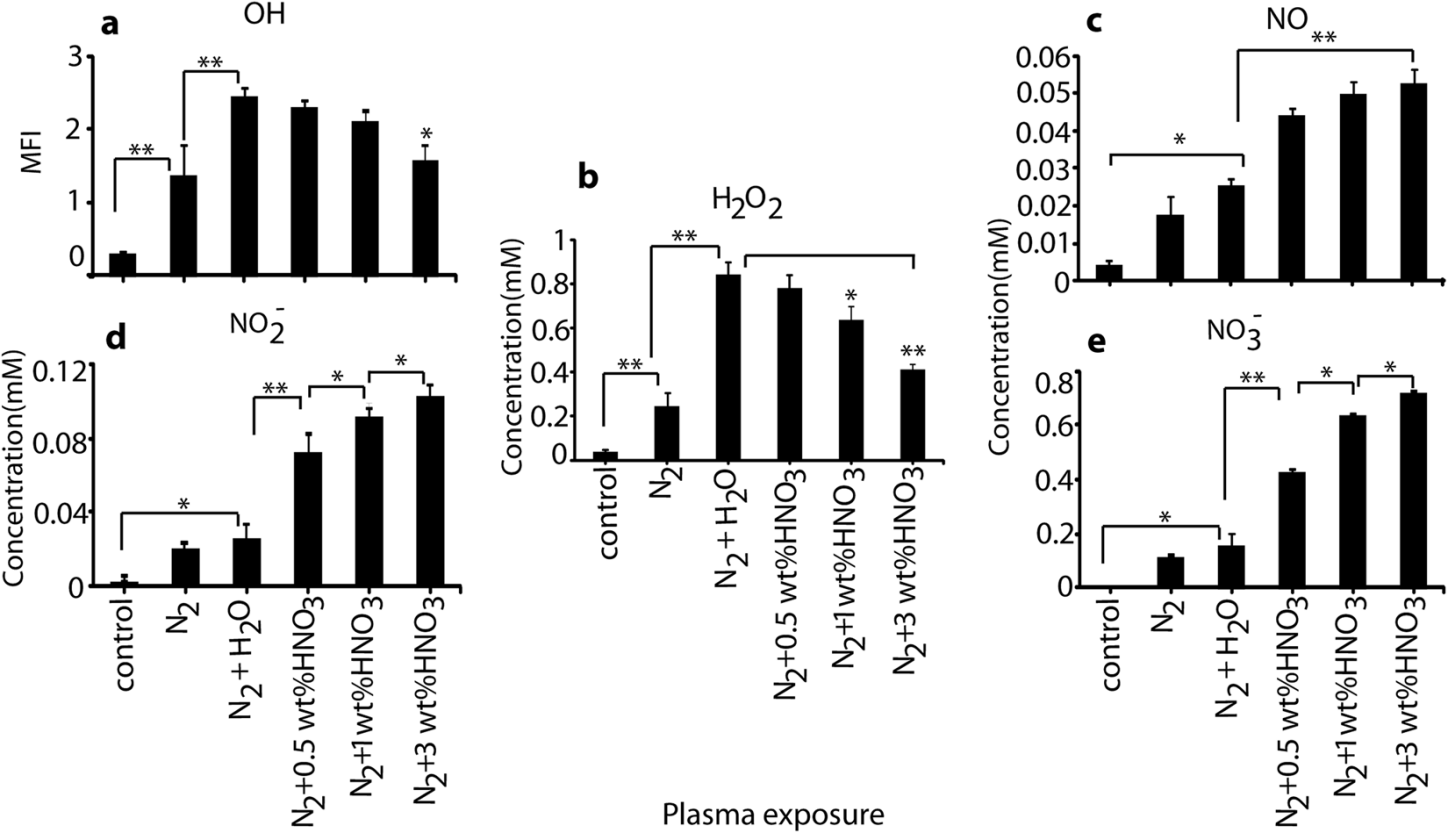
Determination of (**a**) hydroxyl radical; (**b**) hydrogen peroxide; (**c**) nitric oxide; (**d**) nitrite and (**e**) nitrate in DI water after exposure with plasma jet for 10 min, at different conditions (see Fig. 3).

We have also measured the concentration of various RONS generated in the solutions after exposure to plasma at different conditions, i.e., N_2_ (400 ccm) + (0.5, 1 and 3 wt%) HNO_3_ or DI water vapor (in 600 ccm N_2_ gas), and 1000 ccm N_2_ gas without vapor. Figure 4(a) shows that 10 min treatment by N_2_ + H_2_O vapor plasma yields a higher amount of OH species in the solution, compared with N_2_ + (0.5, 1, and 3 wt%) HNO_3_ vapor plasma, and especially compared with the pure N_2_ plasma. The OH radicals are the main source of H_2_O_2_, and Fig. 4(b) indeed illustrates that the N_2_ + H_2_O vapor plasma produced the highest concentration (0.9 mM) of H_2_O_2_, while the N_2_ + HNO_3_ (0.5, 1, and 3 wt%) vapor plasma yielded H_2_O_2_ concentrations of ≈0.8, 0.7 and 0.6 mM, respectively, and the pure N_2_ plasma produced only 0.3 mM of H_2_O_2_. Among the different HNO_3_ vapor plasmas, the 0.5 wt% of HNO_3_ plasma generated the highest H_2_O_2_ concentration.

For NO, we found the opposite results as for the OH radicals and H_2_O_2_, i.e., a higher amount of NO was observed for N_2_ + (0.5, 1, and 3 wt%) HNO_3_ vapor plasma, as compared to the pure N_2_ plasma and the N_2_ + H_2_O vapor plasma, as shown in Fig. 4(c). This is logical, because the HNO_3_ vapor generates more NO upon dissociation, both in gas and liquid phase. For the same reason, NO_2_^−^ and NO_3_^−^ showed similar results: the N_2_ + HNO_3_ vapor plasma (at different wt%) yields higher NO_2_^−^ and NO_3_^−^ concentrations, as compared to the pure N_2_ plasma and the N_2_ + H_2_O vapor plasma, as shown in Fig. 4(d,e). The 3 wt% HNO_3_ vapor system produced the highest amounts of NO, NO_2_^−^ and NO_3_^−^.

Although 3 and 1 wt% HNO_3_ vapor produces more RNS in comparison to 0.5 wt% HNO_3_ vapor, the exposed water at these conditions becomes much more acidic, as shown in Fig. 3a. Hence, to avoid the effect of pH in bacterial inactivation, we have chosen the N_2_ + 0.5 wt% HNO_3_ vapor plasma system for the further research.

### Efficacy of PTW generated with N_2_, N_2_ + H_2_O vapor and N_2_ + 0.5 wt% HNO_3_ vapor plasma in *E. coli* inactivation, and comparison with direct plasma treatment

**Figure 5:**
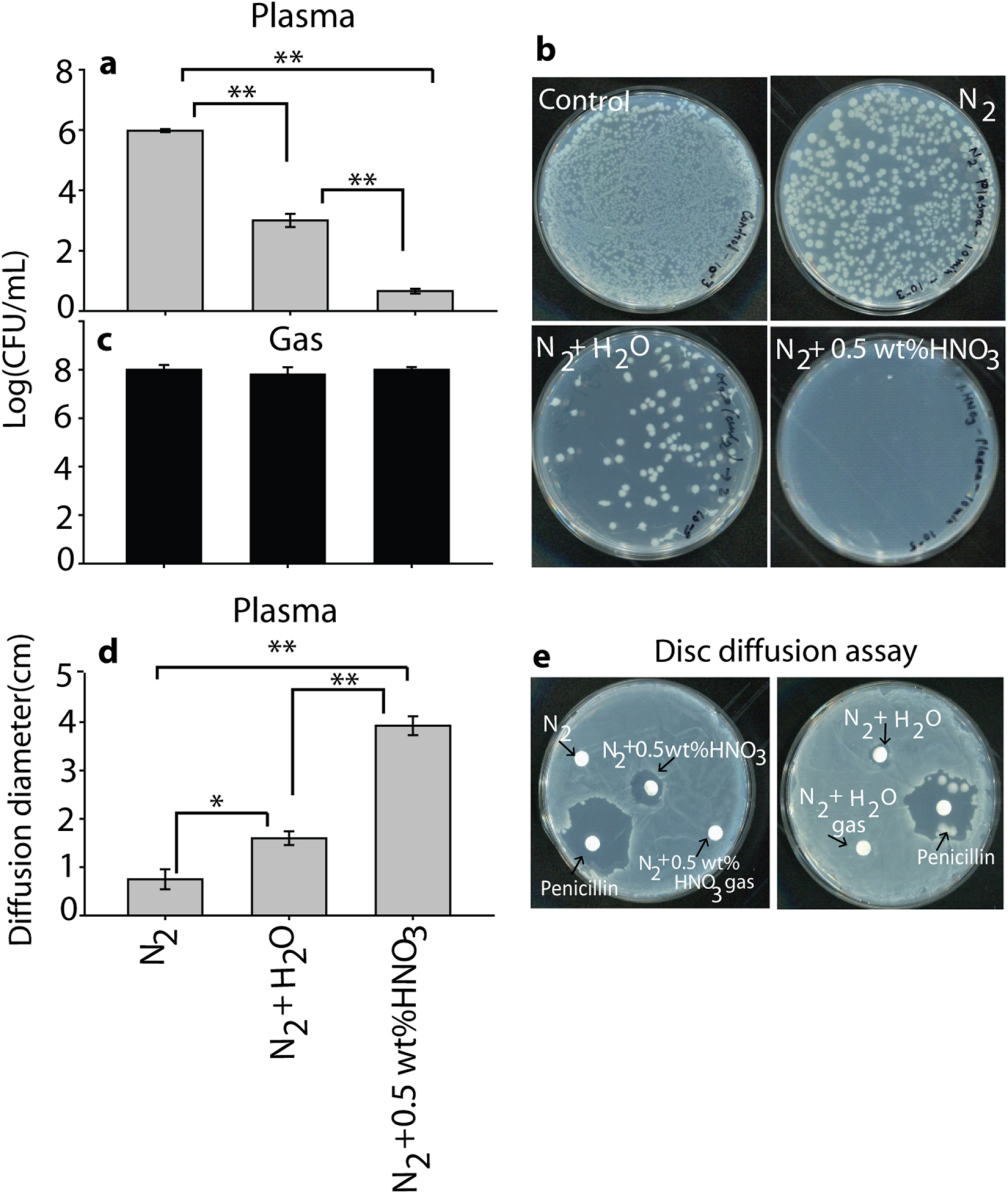
(**a**) Inactivation of *E. coli* after different types of PTW treatments, (**b**) Colony of *E.coli* inactivation after different types of PTW treatments, (**c**) Inactivation of *E.coli* after gas treated water and (**d,e**) Disc diffusion assay after 3 hr incubation with PTW generated by N_2_, N_2_ + H_2_O vapor and N_2_ + 0.5% HNO_3_ vapor.

To determine the efficacy of PTW produced by N_2_, N_2_ + H_2_O vapor, and N_2_ + 0.5 wt% HNO_3_ vapor plasma, we checked the *E. coli* inactivation after 3 h incubation. Figure 5(a,b) show that exposure with PTW generated in case of 0.5 wt% HNO_3_ vapor has a higher effect on *E. coli* inactivation, as its CFU reduces by 5 log values. The pure N_2_ generated PTW reduces the CFU by 2 log values, while the PTW generated by N_2_ + H_2_O vapor reduces the CFU by 4 log values. In comparison, PTW exposed to N_2_, N_2_ + H_2_O vapor, and N_2_ + 0.5 wt% HNO_3_ vapor gas flow (i.e., without plasma) has no effect on *E. coli* inactivation (Fig. 5(c)), which indicates that the effects are clearly due to the RONS created by the plasma. Figure 5(d,e) show similar results in the disc diffusion assay: PTW created by N_2_ + 0.5 wt% HNO_3_ vapor has a higher zone of inhibition, in comparison with N_2_ PTW and N_2_ + H_2_O PTW.

To confirm this result, we also used the standard antibiotics agent (Penicillin) to check the zone of inhibition, and the results reveal that PTW created by N_2_ + 0.5 wt% HNO_3_ vapor has strong ability for zone of inhibition similar to Penicillin but higher as compare to N_2_ + H_2_O PTW. Furthermore, we checked the effect of direct plasma action on *E. coli*. Figure S1 shows that direct treatment by N_2_ + 0.5 wt% HNO_3_ vapor plasma leads to significant inactivation of *E. coli*, in comparison with N_2_ plasma and N_2_ + H_2_O vapor plasma treatments. Hence, both direct and PTW treatment using N_2_ + 0.5 wt% HNO_3_ vapor plasma results in the maximum inactivation of *E. coli*.

### Analysis of oxidative stress related gene expression and disruption of *E. coli* cell morphology

**Figure 6:**
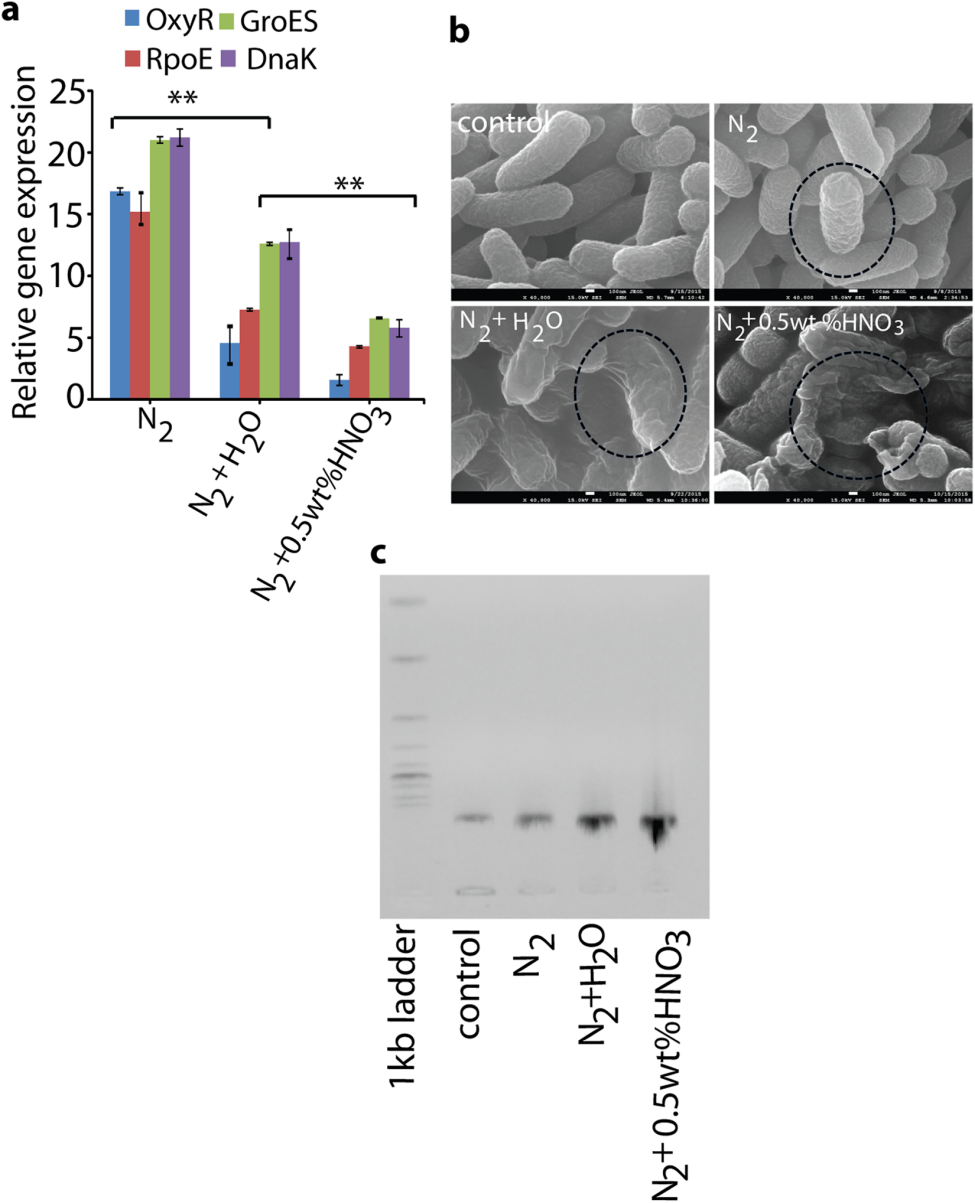
**(a)** Analysis of oxidative related gene expressions, (**b**) cell morphology and (**c**) DNA damage after 3 hr incubation with PTW generated by N_2_, N_2_ + H_2_O vapor and N_2_ + 0.5% HNO_3_ vapor.

To elucidate the mechanism of action of PTW on *E. coli* inactivation, we analyzed the oxidative related gene expression and cell morphology. For the gene expression analysis, we chose four oxidative stress genes, i.e., OxyR (Oxygen regulated gene), RpoE (DNA-dependent RNA polymerase), GroES (Heat-shock gene), and DnaK (Chaperone protein DnaK). These proteins regulate under stress conditions, and help to protect the cells through antioxidant defense mechanisms^35–38^. OxyR and DnaK genes relates to oxidative stress. GroES and DnaK genes relates to the cellular homeostasis^39,40^. The failure of the antioxidant defense machinery under high oxidative stress may lead to inhibitory effects of these gene expressions (OxyR, DnaK, GroES and DnaK) that results in DNA damage. Figure 6(a) shows that 3 h incubation of PTW generated by N_2_ + H_2_O vapor or 0.5 wt% HNO_3_ vapor caused higher inhibitions of the gene expression as compared with N_2_ generated PTW. In contrast, PTW generated by N_2_ + H_2_O or 0.5 wt% HNO_3_ vapor shows inhibition of these gene expressions, because the oxidative stress is above the threshold under these PTW treatments, resulting in collapse of the antioxidant defense machinery.

We also determined the effects of PTW on the morphology of *E. coli*. Figure 6(b) shows that PTW generated by N_2_ + H_2_O vapor and by N_2_ + 0.5 wt% HNO_3_ vapor has crumpled the cells to a greater extent than N_2_ PTW. Finally, we checked the genomic DNA damage through 1% gel electrophoresis. Figures 6(c) and S2 shows that PTW generated by N_2_ + H_2_O vapor and N_2_ + 0.5 wt% HNO_3_ vapor have more effect on the DNA degradation/oxidation.

## Discussions

It is generally known that RONS generated from an APPJ induce inactivation of microorganisms, as well as structural changes in proteins^5,32^. In this work, we demonstrate that the adding a vapor system (either water vapor or 0.5 wt% of HNO_3_ vapor) to the plasma enhances the antimicrobial activity as compared to plasma without vapor system. This is attributed to the increase in ROS and RNS content. The H_2_O vapor system increases the ROS in the solution, but the RNS formation does not increase, whereas the HNO_3_ vapor system can increase both the RNS and ROS content in solution. We analyzed the NO and OH species by optical emission spectrometry in the gas phase and by chemical analysis in the solution, after 10 min plasma treatment. The NO radical concentration dramatically increases inside the solution for plasma treatment with N_2_ (400 ccm) + 0.5 wt% HNO_3_ vapor (600 ccm N_2_ gas), as compared to pure N_2_ plasma treatment or treatment by N_2_ + H_2_O vapor. At this condition, both the NO and OH concentrations increase in the gas phase, as well as inside the solution. On the other hand, for the water vapor system, the OH content increases by a large amount, but the NO concentration did not increase in solution, as shown in Fig. 4.

We measured the transcriptional level of these genes (OxyR, RpoE, GroES, and DnaK) in PTW-treated *E. coli*. When *E. coli* was treated with N_2_ PTW, the transcriptional-related gene expressions of OxyR, RpoE, GroES, and DnaK were enhanced (Fig. 6(a)). In contrast, the N_2_ + H_2_O or 0.5 wt% HNO_3_ PTW showed inhibitory effects on the gene expression in comparison with the control sample. Furthermore, N_2_ + 0.5 wt% HNO_3_ PTW showed higher inhibition effect than N_2_ + H_2_O PTW, which can be explained by the higher RONS content. This study clearly shows that PTW generated by N_2_ + H_2_O vapor or 0.5 wt% HNO_3_ vapor plasma has higher RONS contents, which lead to bacteria killing through inhibition of the antioxidant machinery, which damages the membrane protein repair chaperone, as well as DNA repair cascade^41,42^. Furthermore, we analyzed the DNA degradation through gel electrophoresis and the bacteria morphology, after treatment with PTW generated by all plasma systems. Among all systems (N_2_ plasma, N_2_ + H_2_O vapor plasma, and N_2_ + 0.5 wt% HNO_3_ vapor plasma), the PTW generated by N_2_ + 0.5 wt% HNO_3_ vapor plasma shows more *E. coli* deactivation.

This study demonstrates that PTW produced from N_2_ + H_2_O vapor plasma and N_2_ + 0.5 wt% HNO_3_ vapor plasma systems has a high antibacterial efficiency. According to literature, NO_2_^−^ and H_2_O_2_ are important for the bactericidal activity^21^. At our experimental conditions, the N_2_ plasma produces ≈0.02 mM NO_2_^−^ and ≈0.3 mM H_2_O_2_, while the N_2_ + H_2_O vapor plasma produces ≈0.025 mM NO_2_^−^ and ≈0.9 mM H_2_O_2_, and the N_2_ + 0.5 wt% HNO_3_ vapor plasma produces ≈ 0.076 and 0.8 mM of NO_2_^−^ and H_2_O_2_, respectively. Thus, the maximum NO_2_^−^ and H_2_O_2_ concentrations are produced by the N_2_ + 0.5 wt% HNO_3_ vapor plasma and the N_2_ + H_2_O vapor plasma, respectively. If we compare the bactericidal activity in Fig. 5, we observe the maximum deactivation of bacteria for the PTW generated by the N_2_ + 0.5 wt% HNO_3_ vapor plasma, followed by the N_2_ + H_2_O vapor plasma, and the smallest effect for the N_2_ plasma PTW. This shows that a high concentration of both NO_2_^−^ and H_2_O_2_ plays a key role in sterilization. It was reported previously that the presence of H_2_O_2_ and HNO_2_ produces ONOO^−^, which is the main component for inactivation of bacteria^28^. However, ONOO^−^ has a short lifetime in acidic solution, and therefore it cannot be the main component of PTW for the antibacterial activity^26,30^. On the other hand, Ikawa *et al*. reported that O_2_^•−^ is mainly responsible for the bactericidal activity^31^. The authors also claimed that the production of O_2_^•−^ does not require the presence of oxygen, as it is generated by O_2_NOOH. They also suggested that H_2_O_2_ is the main component for the production of O_2_NOOH in PTW along with NO_2_^−^. Therefore, we may conclude that the N_2_ + 0.5 wt% HNO_3_ vapor plasma produces the highest amount of NO_2_^−^ and H_2_O_2_ in PTW, which can form O_2_NOOH by multiple reactions, and this further leads to the formation of O_2_^•−^ which contributes to sterilization. On the other hand, the N_2_ + H_2_O vapor plasma produces a high amount of H_2_O_2_ but a lower content of NO_2_^−^; and this results in a lower production of O_2_^•−^, and thus in a lower antibacterial activity. Additionally, the pH of PTW after N_2_ + 0.5 wt% HNO_3_ vapor plasma and N_2_ + H_2_O vapor plasma is near 4.5, and it was reported that the half-life of O_2_NOOH is 1.6 min at pH 4.7^43^. Hence, we believe that in our study O_2_NOOH is the main factor for the bactericidal activity through O_2_^•−^ formation.

## Conclusion

We studied the antibacterial effect of PTW generated by various plasma systems, i.e., N_2_ plasma, N_2_ + H_2_O vapor plasma, and N_2_ + 0.5 wt% HNO_3_ vapor plasma, and we clear demonstrated that the vapor plasma, and especially the N_2_ + 0.5 wt% HNO_3_ vapor plasma, was more efficient for the deactivation of bacteria than PTW generated by plasma without vapor system. The results of this research support the idea that a high concentration of both NO_2_^−^ and H_2_O_2_ is important for the antibacterial activity, through the creation of other RONS, such as O_2_NOOH and O_2_^•−^.

## Electronic supplementary material

Supporting information
