# Supplementary material for: Bacterial inactivation by plasma treated water enhanced by reactive nitrogen species
Source: Sci Rep. 2018 Jul 26;8:11268. doi: 10.1038/s41598-018-29549-6 (PMC6062550; doi:10.1038/s41598-018-29549-6)
Supplement: Supplementary file 1 — Supporting information [file 41598_2018_29549_MOESM1_ESM.doc]

**Supporting Information**

**Bacterial inactivation by plasma treated water enhanced by reactive nitrogen species**

Priyanka Shaw^§a,b^, Naresh Kumar^§a,b^, Hyong Sin Kwak^a^, Ji Hoon Park^a^, Han Sup Uhm^a^, Annemie Bogaerts^b^, Eun Ha Choi^a*^ and Pankaj Attri^a,b*^

*^a^Plasma Bioscience Research Center/ Department of Electrical and Biological Physics, Kwangwoon University*, *20 Kwangwon-Ro*, *Nowon-Gu, Seoul 139-701, Korea*

^b^ *Research group PLASMANT, Department of Chemistry, University of Antwerp, BE-2610 Wilrijk-Antwerp, Belgium*

**Fig. S1**: Direct plasma action on E. coli, based on N_2_ plasma, N_2_ + H_2_O vapor plasma and N_2_ + 0.5 wt% HNO_3_ vapor plasma.

**Fig**. **S2**: Full image of DNA damage after 3 hr incubation N_2_, N_2_+H_2_O and N_2_ + 1% HNO_3_ PAW.

**Fig. S1**


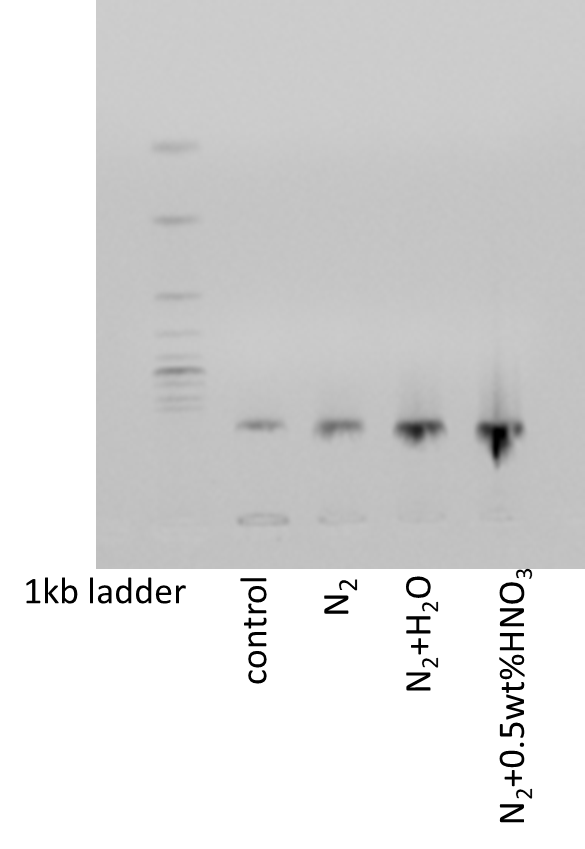


**Fig. S2**
